# Evaluation of microfiber release from jeans: the impact of different washing conditions

**DOI:** 10.1007/s11356-021-14761-1

**Published:** 2021-06-11

**Authors:** Aravin Prince Periyasamy

**Affiliations:** Textile Chemistry, Department of Bioproducts and Biosystems, Aalto University, Espoo, 02150 Finland

**Keywords:** Microplastics, Microfibers, Jeans, Textiles, Domestic washing

## Abstract

Microplastic particles are a burgeoning population crisis in the marine environment. This research examines the emission of microfibers from three different jeans (garments) during domestic washing. The jeans types, washing temperature, washing duration, spin speed, detergent types, and addition of conditioner are the main factors for this research work. The average length and diameter of the microfibers for the 100% PET jeans (jeans-P) has 7800 ± 4000 μm and 11.9±3.2 μm and for polyester/cotton jeans (jeans-PB) has 4900 ± 2200 μm 17.4±4.8 μm, respectively. The maximum microfiber released was observed in the rigorous washing treatment (90 min, 60°C, 1400 rpm, powder detergent with the presence of conditioner). The surmised number of microfibers discharged from the 1 kg wash load of jeans-P was calculated within the extent of 2300000–4900000 microfibers, and it is varied by the washing treatments.

**Supplementary Information:**

The online version contains supplementary material available at 10.1007/s11356-021-14761-1.

## Introduction

Microplastics have contaminated the environment including water sources such as the ocean and the lakes (Browne et al. 2011; Thompson 2013). Microplastics are synthetic solid particles that have different forms which include microflakes, microbeads, and microfibers (Dervishi 2019). The sources of microfibers in the textile industry are clothing as well as non-clothing applications such as mattresses (Thompson and Olse 2004; Wagner et al. 2014). Generally, these microfibers are observed in both freshwater and marine environment (Neves et al. 2015). It is reported that microplastics are present in the aquatic habitats, beaches, surface waters, and subtidal sediments in a wide range and their abundance is substantially increasing (Thompson and Olse 2004). Barrett et al. (Barrett et al. 2020) estimate at least 14 million tonnes of plastic particles with less than 5 mm accumulated in the bottom of the ocean. Huge quantities of microplastics were found in non-industrialized places like Niue, Arutanga, Fiji (Gregory 2009; Gross 2015). The potential effects in the environment will be influenced by the microplastics which contain two types of chemicals: (i) monomer, polymer, and other additives including colorants and (ii) chemicals absorbed from the surrounding (Campanale et al. 2020). Therefore, it is important to recognize the relative abundance and the sources of various types of microplastics (Koelmans et al. 2013; Bakir et al. 2014). In this work, the shedding of micro-sized fibers from the garments during domestic washing (1 to 5mm) is referred to as microfibers. Jeans are the most fashionable and widely used garment (Barajas 2013; Periyasamy et al. 2017) among youngsters. In recent decades, the market potential for jeans has increased tremendously and it is expected to grow by $3.53 bn during 2020–2024 (Technavio 2020). Approximately, 1.9 billion pairs of jeans were sold alone in 2015, which is expected to grow 2.2 billion by 2021 (Technavio 2019). A range of textile fibers was used to produce jeans; polyester (PET) is one among them (Kan et al. 2010, 2011).

Due to the population explosion, the amount of microplastics released to the environment rapidly increases (Ritchie and Roser 2018; Chen et al. 2020; Schernewski et al. 2020). Browne et al. (Browne et al. 2011) studied and suggested that the highest source of microplastic in the form of microfibers is obtained from domestic washing. To evaluate the number of microfiber shedding during domestic washing, various approaches have been developed (Browne et al. 2011; Pirc et al. 2016; Napper and Thompson 2016; De Falco et al. 2018b, c, 2019). Particularly, it is observed that the garments made from PET, acrylic, and polyester/cotton at two temperatures (30°C and 40°C) in the presence/absence of a detergent and a fabric conditioner (Napper and Thompson 2016), in this work, a garment made from acrylic emits 728789 microfibers per 6 kg of garments. Also, 0.0012 wt.% of microfibers are released from PET fleece blankets for each washing process determined by a similar approach (Pirc et al. 2016). In other work (De Falco et al. 2019), the synthetic clothes release which ranges between 124 and 308 mg for 1 kg of washed clothes vary according to the type of clothes. In other works, the shedding of microfibers released purely depends on the washing conditions, in particular detergents play a vital role (Napper and Thompson 2016; Hernandez et al. 2017; De Falco et al. 2018a). Washing additives like softeners can reduce the microfiber generation up to 35% (De Falco et al. 2018c). The aged fabrics generally release higher microfibers than the new fabrics (Hartline et al. 2016). Additionally, the number of  microfibers released also depends on the washing machines (top-versus and front-load) (Hartline et al. 2016) being utilized, as it is beyond the scope of this study, it and has not been carried out and will be accomplished in near future.

To estimate the real environmental impact of domestic washing of synthetic jeans is essential. It is necessary to identify the parameters which influence the release of microfibers during the washing process. Various works have been published on this topic where the comparisons are not clear due to different methodologies adopted. Most of these works performed on the different synthetic fabrics and their structures (Napper and Thompson 2016; Hartline et al. 2016; Sillanpää and Sainio 2017; De Falco et al. 2018a, 2020; Stanton et al. 2019). To our knowledge, there is no research carried out on the different compositions of jeans with varied washing conditions. An investigative study has been conducted to account the quantity and dimension of microfibers released during washing of three different (i.e., 97% PET + 3% elastane; 70% PET + 27% cotton + 3% elastane, and 50% PET + 50% cotton) jeans. In this work, the washing treatments are varied such as detergent types, the addition of conditioner, washing temperature (30°C, 45°C, and 60°C), washing duration (60, 75, and 90 min), and spin speed (1200 and 1400 rpm). The jeans types, washing temperature, washing duration, spin speed, detergent types, and addition of conditioner are the main factors for this research work since these factors strongly influence the microfiber generations.

## Materials and methods

For this investigation, branded jeans (bottoms) with three different compositions were purchased from fashion outlets in Coimbatore, India. While purchasing, different color of jeans has been chosen to readily differentiate the emitted fibers. The basic properties of three different jeans are given in Table 1. The detergents (both powder and liquid) and citric acid were purchased from the local shop and Sigma Aldrich India, respectively. The image of washed and unwashed jeans is shown in Figure S1.

**Table 1:** Physical properties of jeans used for this study

Composition		97% PET + 3% Lycra	70% PET + 27% Cotton + 3 % Lycra	50% PET + 50% cotton
Abbreviation		jeans-P	jeans-PA	jeans-PB
Cover factor (Yarns/inch)	warp	81	83	84
	weft	51	51	49
Hairiness index	warp	4.3	3.8	3.8
	weft	4.3	3.8	3.8
Staple fiber length (mm)		38	32	32
Linear density (tex)	warp	59/1	59/1	60/1
	weft	36.8/1	42/1	40/1
Construction		3x1 RHT	3x1 RHT	3×1 RHT
Style		Women’s bottom	Women’s bottom	Women’s bottom
Color		Blue	Light blue	Navy
Single garment weight (grams)		453	457	462

Note: *RHT* right-hand twill

### Washing process

For the washing process of jeans, the Whirlpool FRESH CARE has been used. The washing machine is brand new with front-loading technology. There is no other reason for selecting this washing machine rather than the availability as well as the popularity in India. Prior to the garment washing, the machines were cleaned twice by using citric acid with rigorous settings (120min, 60°C, 1400 rpm). A single garment (jeans) was washed separately and dried in atmospheric conditions (i.e., line dry); later, the garments were worn to simulate the real wash (i.e., home laundering) and wear conditions. The washing was carried out by implementing six levels (A–F) of detergent/conditioner treatments × washing duration with three levels (60, 75, 90 min) × washing temperature with three levels (30, 45, 60°C) × spin speed with two levels (1200, 1400 rpm), and thus, there were 108 (6 × 3 × 3 × 2 = 108) experiments. To reduce the statistical error, after wearing, each garment was washed four times (i.e., replicates) separately according to the specific washing conditions (i.e., 108 × 4 = 432), for all the jeans used for this work (jeans-P, jeans-PA, and jeans-PB). The detailed information on the washing conditions is given in Table 2. The quantity of conditioner and detergents had been kept constant throughout the cycles with the same volume of water (15 L). This research is to investigate the microfibers released in the washing machine which does not contain the tumble dryer since such machines are expensive and not commonly used in India. Usage of a tumble dryer after washing is beyond the scope of this present study. The inclusion and exclusion criteria for washings were provided in Table S1 and the composition of different detergents is given in Table S2.

**Table 2:** Detailed information on the washing conditions uses for this work

Abbreviation (six levels)	Washing dose (for 15 L water)	Washing duration (min)			Washing temperature (°C)			Spin speed (rpm)	
A	No detergent + no conditioner	60	75	90	30	45	60	1200	1400
B	No detergent + conditioner (40 mL)	60	75	90	30	45	60	1200	1400
C	Liquid detergent (60 mL) + no conditioner	60	75	90	30	45	60	1200	1400
D	Liquid detergent (60 mL) + conditioner (40 mL)	60	75	90	30	45	60	1200	1400
E	Powder detergent (65 g) + no conditioner	60	75	90	30	45	60	1200	1400
F	Powder detergent (65 g) + conditioner (40 mL)	60	75	90	30	45	60	1200	1400

### Filtration, removal, and counting of the microfibers

The total quantity of washing effluent was collected from the washing machine after the washing process of single jeans. The residual microfibers were collected by filtering the wastewater in the external filters including stainless steel (200 × 200 μm), and PTFE (5 μm) was fixed at the end of the drain hose. Once the washing is completed, the filter was removed carefully, dried in atmospheric condition. While drying, both filters were covered by aluminum foil to avoid airborne. Later, the microfibers were collected cautiously to weigh it from both stainless steel and PTFE filter. The emitted microfibers per jeans are calculated based on the following equation (Napper and Thompson 2016; De Falco et al. 2018b).

1 $$ T=\left(\frac{4{A}_{MP}}{\pi {D}^2 l\rho}\right) $$

where *T* is the total number of microfibers per jeans, and *A*_*MP*_ is the total mass of microfibers collected from both filters. Assuming that the collected fibers were cylindrical structure, (Napper and Thompson 2016), *D* and *l* are the average diameter and length of extracted fibers, *ρ* is the density of the material (for PET-1.38 g/cm^3^, for lycra 1.25 g/cm^3^, and cotton is 1.540 g/cm^3^).

### Characterization

After drying, the microfibers were carried out to analyze the surface morphology. Both, scanning electron microscope (SEM) and the optical microscope were used for the surface analysis. TS5130 Vega-Tescan SEM (Czech Republic) was used with the following conditions: 20 kV accelerating voltage and ×500 with a vacuum of 7.8×10^−3^ Pa. OLYMPUS SZ61 optical microscope was used for the complement micrographs on the microfibers to evaluate the length and diameters of the fibers. Later, the SEM images were analyzed by using public domain software ImageJ in order to measure the length and diameter of the microfibers collected from both the filters; it can be repeated 40 times to reduce the statistical error, and the mean values are used for further calculations. The working distance between the sample and the objective lens can be adjusted according to the image resolution. For the confirmation of residual fibers’ chemistry, the Fourier transform infrared spectroscopy (FTIR-ATR) was used before and after the washing process. Statistical analysis was conducted using IBM SPSS 26 (IBM, Inc., Armonk, NY, USA). A 5-way analysis of variance (ANOVA) was analyzed between the jeans types, different washing treatments, spin speed, washing duration, and washing temperature. The criterion for statistical significance was set as 0.05.

## Results and Discussions

### Characterization of microfibers

**Fig. 1:**
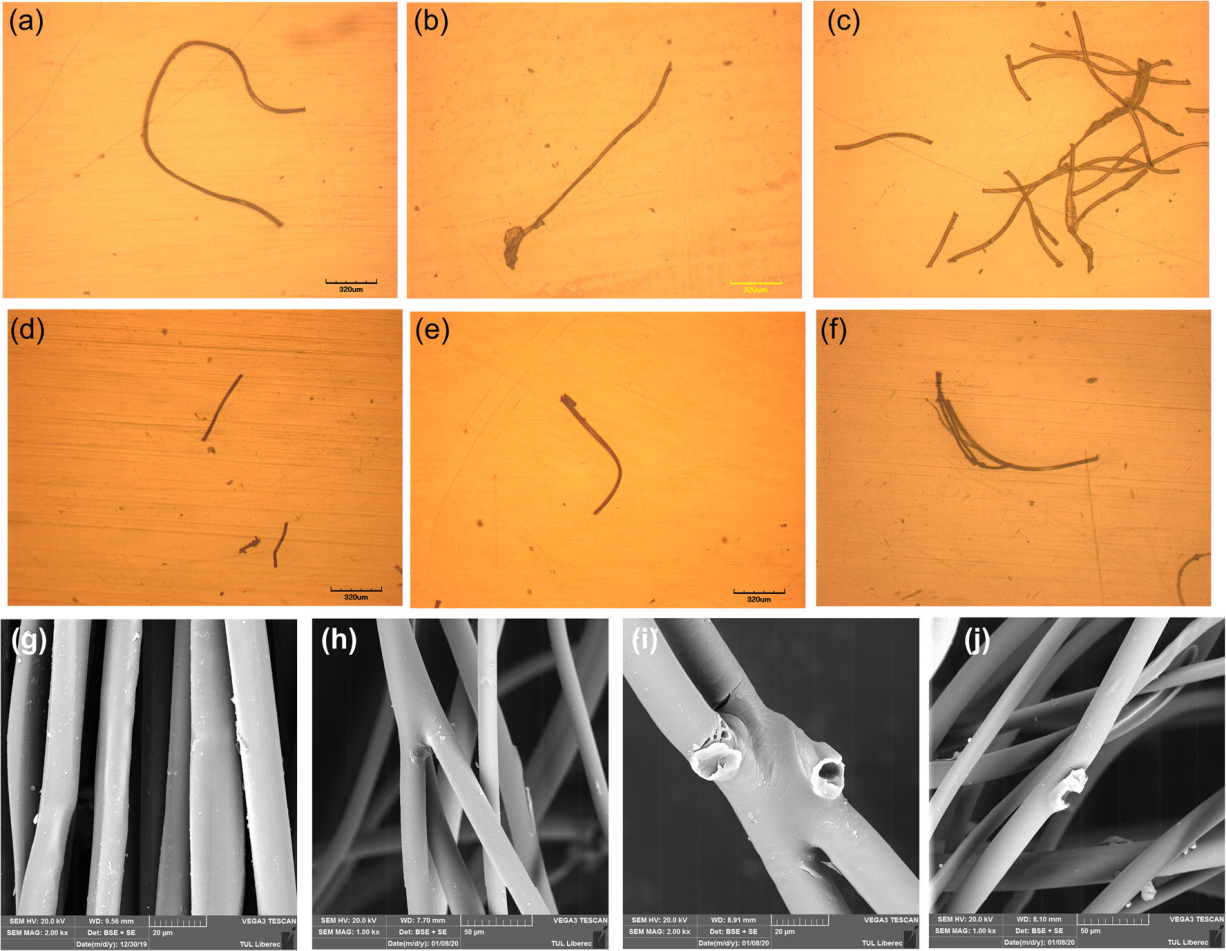
Optical microscopy images (×100) of representative fibers collected on the 5 μm PTFE filter, jeans-P (**a**–**c**); jeans-PB (**d**–**f**); SEM images of jeans-P unwashed (**g**); washed (**h**–**j**)

The morphological analysis for the microfibers collected from the washing effluent has been analyzed, and the images are shown in Fig. 1. From this image, it is evidenced that several fibers show the surface damage or ripped-off from the fiber structure (Fig. 1b, j); also, there is a fracturing of the fiber in a few situations (Fig. 1h). However, a few fiber fragments show surface damages due to the washing actions (Fig. 1b, c, e, and f). This is due to the consequence of mechanical stress suffered by the jeans garment throughout the washing process. Besides, the detachment of microfibers was observed as it is clearly shown in Fig. 1e, i, and j. Further, SEM images were used to calculate the average length of collected microfibers from the washing effluent. In some cases, it is very difficult to measure, due to the entanglement of fibers in which some are strongly bound together (i.e., in particular, the microfibers collected from higher spin speed and higher washing temperature). Therefore, the average length of the microfibers estimation was under-estimated. However, the measurement takes 40 different locations of fibers to calculate the average length and diameter. The average length and diameter of the microfibers released from different types of jeans were tabulated in Table 3.

**Table 3:** Details of microfiber generations in different jeans

Factors	jeans-P	jeans-PA	jeans-PB
Length (μm)	7800 ± 4000	6660 ± 4000	4900 ± 2200
Diameter (μm)	11.9 ± 3.2	12.6 ± 4.2	17.4±4.8
Microfiber releases per kg of jeans	2305395–4874323	619581–1085431	561683–865182

### ATR-FTIR spectra for microfibers

To evaluate the chemistry of microfibers collected after the washing process, ATR-FTIR spectroscopy was utilized to characterize the functional groups with the resolution of wavenumber range from 400 to 4000 cm^−1^. The spectra for jeans (before washings) and released microfibers (after washings) can be compared and concluded with the help of the Institute of chemical University of Tartu, Estonia (Peets et al. 2017) guidelines. Both before and after washings were observed with similar peaks for all the types of jeans (i.e., jeans-P, jeans-PA, and jeans-PB). A detailed description of the observed peaks was given in Figure S2.

### Domestic washing and microfibers emission

The quantity of microfibers released is determined and plotted in Figs. 3, 4, and 5.

#### Impact on washing Temperature

**Fig. 2:**
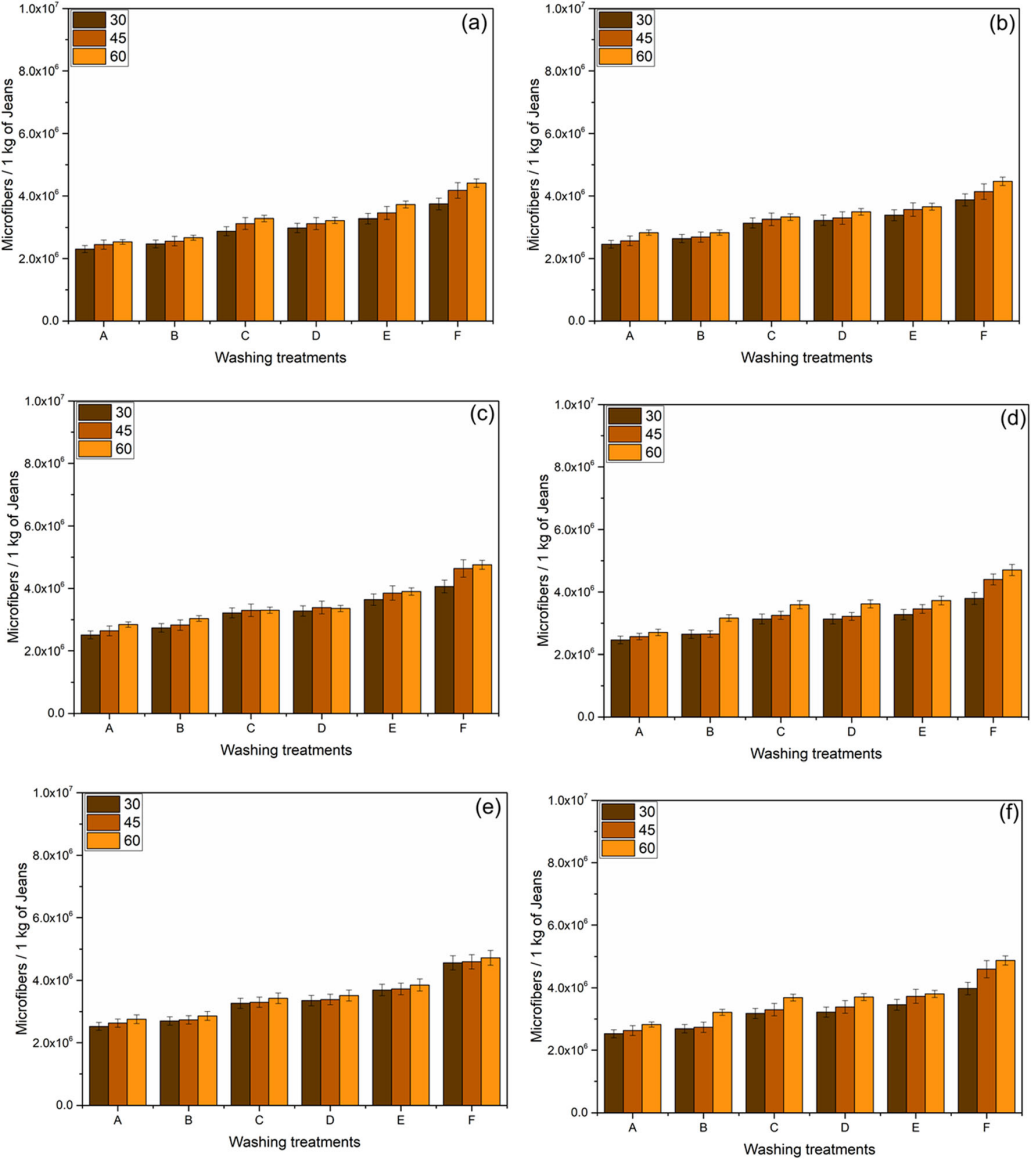
An average relative of microfibers released during successive washing of jeans-P, 60 min washing time spin speed 1200 rpm (**a**); 75 min washing time spin speed 1200 rpm (**b**); 90 min washing time spin speed 1200 rpm (**c**); 60 min washing time spin speed 1400 rpm (**d**); 75 min washing time spin-speed 1400 rpm (**e**); and 90 mins washing time spin speed 1400 rpm (**f**) (error bars represent standard deviation)

**Fig. 3:**
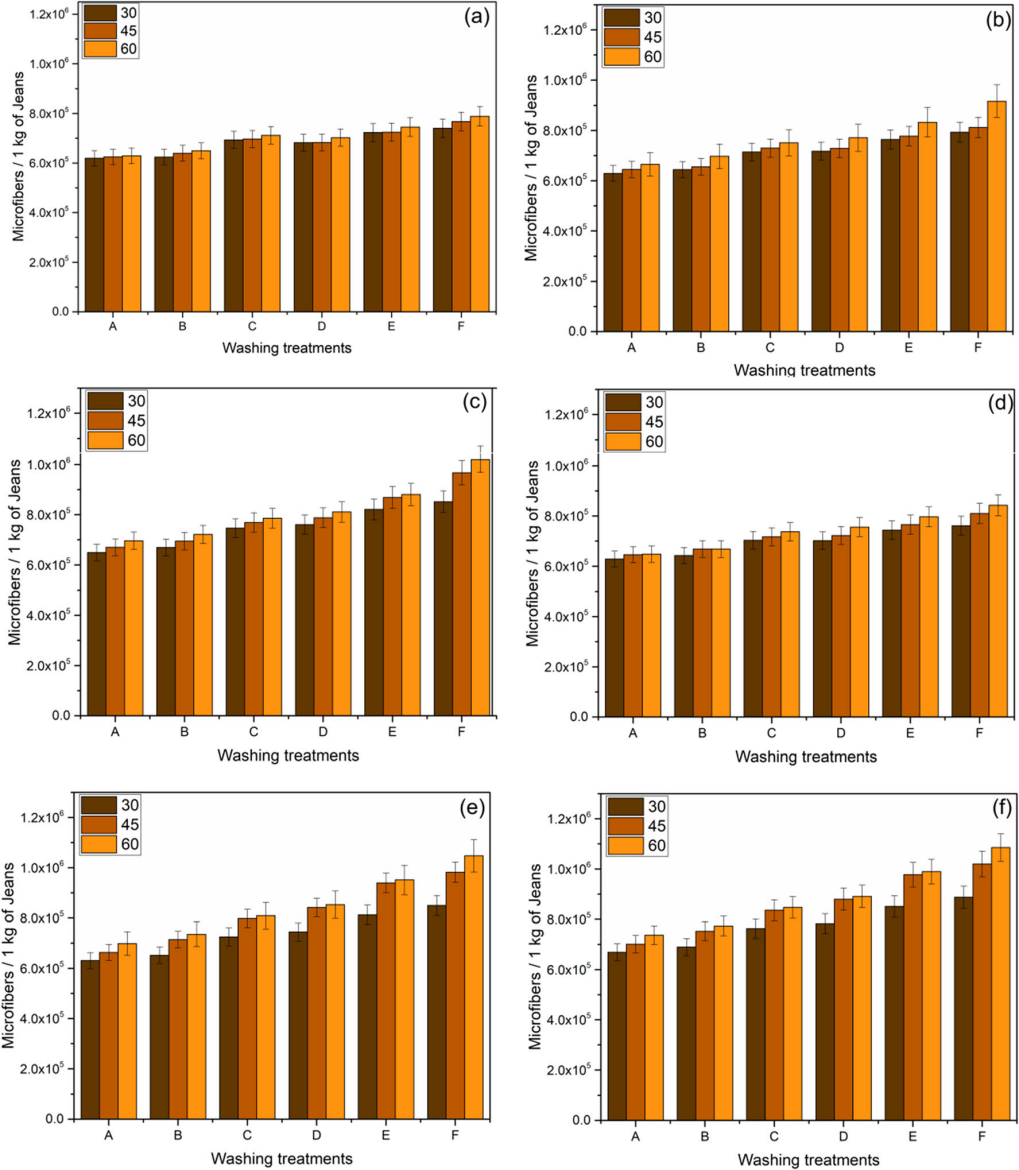
An average relative of microfibers released during successive washing of jeans-PA, 60 min washing time spin speed 1200 rpm (**a**); 75 min washing time spin speed 1200 rpm (**b**); 90 min washing time spin speed 1200 rpm (**c**); 60 min washing time spin speed 1400 rpm (**d**); 75 min washing time spin speed 1400 rpm (**e**); and 90 mins washing time spin speed 1400 rpm (**f**) (error bars represent standard deviation)

**Fig. 4:**
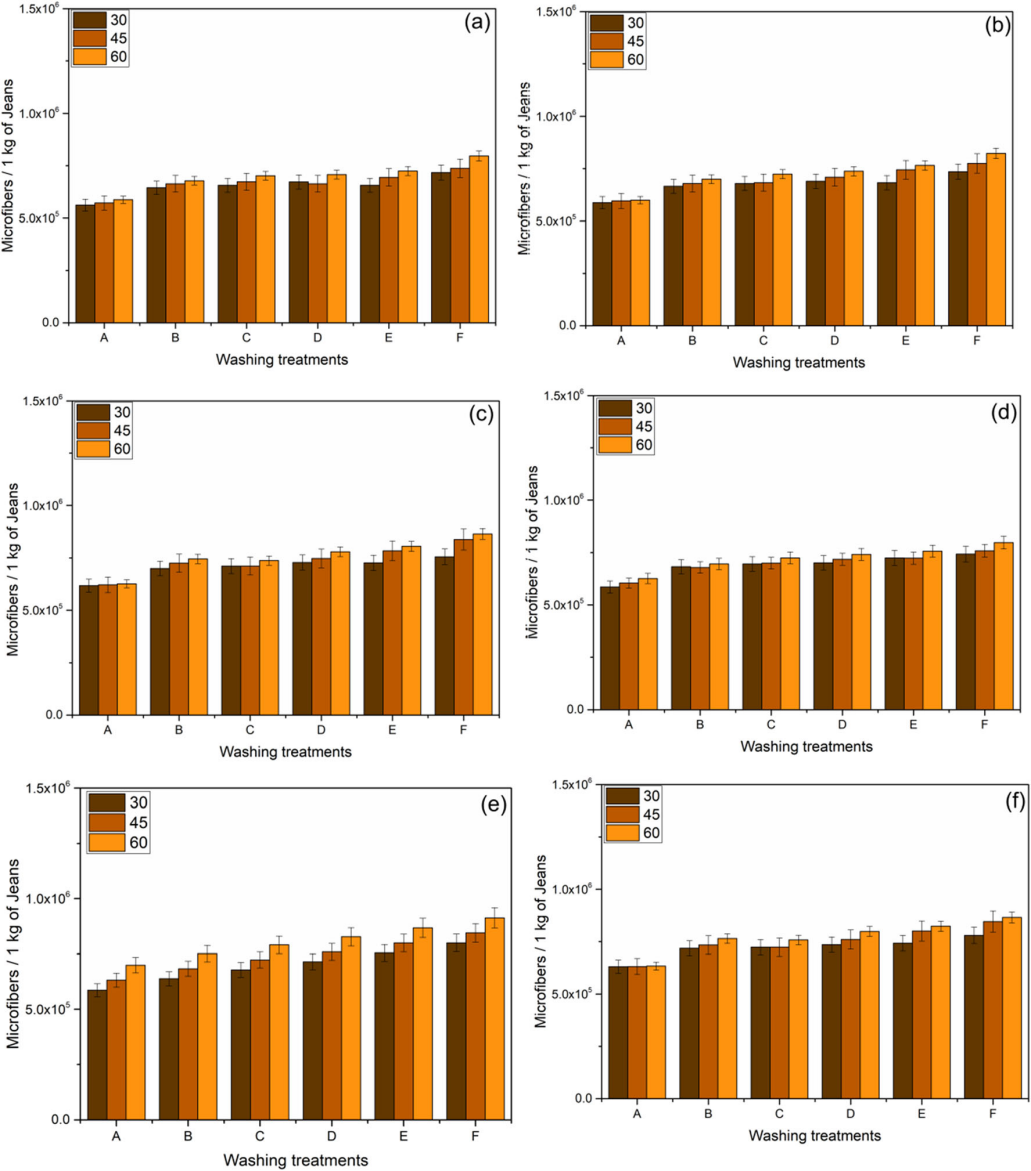
An average relative of microfibers released during successive washing of jeans-PB, 60 min washing time spin speed 1200 rpm (**a**); 75 min washing time spin speed 1200 rpm (**b**); 90 min washing time spin speed 1200 rpm (**c**); 60 min washing time spin speed 1400 rpm (**d**); 75 min washing time spin speed 1400 rpm (**e**); and 90 min washing time spin speed 1400 rpm (**f**) (error bars represent standard deviation)

In the jeans-P, maintaining the constant washing treatment “C,” 2877278, 3121692, and 3283341 of microfibers were released varying with the washing temperature from 30°C, 45°C, and 60°C, respectively (Fig. 2); the observed trend is similar for all washing treatments. Temperature is directly proportional to the release of microfibers which is depicted in (Figs 2, 3, and 4). By maintaining the temperature constant, 6.9%, 21.4%, 21.4%, 24.8%, and 35% of microfibers are recorded with the washing treatments of B, C, D, E, and F, respectively (Fig. 2d). The release of microfiber increases with the increasing washing temperature irrespective of the fabric used. Evidence shows that the possibility of jeans-P to release higher microfibers during washing with high temperature is due to the surface hydrolysis characteristic of polyester under alkaline medium (i.e., powder detergent). On the other hand, temperature increases the swelling action of the cellulose portion in the jeans under alkaline condition and also increases (Duckworth and Wrennall 1977; Nishimura and Sarko 1987) the fuzz formation. The hydrogen bonds between the cellulose chains are disrupted when the detergent mixed with water pierced into the amorphous regions of the cellulose fibers resulting in the fiber swelling. Henceforth, the cellulosic fibers swell as the temperature increases consequently creating free space in the textile structure for the mobilization of the broken fibers. Fuzz formation on the surface of swollen fibers is increased due to the mechanical actions in the washing process (Fig. 6). It further increases with increasing the washing temperature, spin speed, and duration.

#### Impact of washing detergent

When the garments are washed using a surfactant which creates the foam, it results in a reduction of mechanical agitation during the washing process (Coons et al. 1987; Bishop 1995; Sommer 2001). Yet, similar results were not observed in our experiment as the release of microfibers is higher by adding the detergent as well as conditioner. Our investigation emphasizes the results that the microfibers are abundant due to the addition of detergents. Washing treatment “B” for jeans-P releases (i.e., spin-speed of 1200 rpm, 30°C with 75 min) 6.85%, 21.8%, 23.7%, 31.3%, and 36.4% higher microfibers on the treatments of B, C, D, E, and F, respectively (Fig. 2b) as compared to the washing treatment “A.” There is a similar trend observed in Figs. 3b (jeans-PA) and 4b (jeans-PB). The release of microfibers is higher when powder detergent (i.e., washing treatment “E”) was used; this is due to the alkalinity of detergent (i.e., the pH of powder and liquid detergent is 10.6 and 9.1, respectively) as well as the presence of inorganic compounds (TiO_2_ dyes). The alkaline nature of the powder detergent causes surface hydrolysis of jeans-P when exposed to higher washing temperatures and washing time. Powder detergent also induces chemical damage to the jeans which is exposed to the extended washing time. The hydrolysis of PET fibers takes place when the PET undergoes a hot alkaline environment, which causes the nucleophilic substitution reaction on hydroxyl ion which attacks the carboxyl carbon of PET following the chain scission resulting in the production of hydroxyl and carboxylate end groups (Dave et al. 1987; Zeronian and Collins 1989). Additionally, the friction between the jeans and machine drum increases due to the inorganic compounds present in the powder detergent. Apart from that, poor alkali fastness of indigo dye-fiber bonds (Chakraborty 2014; Sarkar 2015), resulting in the bond breakage and releasing microfibers in jeans-PA and jeans-PB. Both the liquid and powder detergent induce the increase of microfiber releases which is based on these results as compared to the washing treatment “A.” That is the reason why the powder detergent has a strong influence on the shedding of microfibers from all types of jeans during the washing (Figs. 2, 3, and 4). In this study, the surface of the filter shows no accumulation or thick layer formation as the detergent dissolved completely as found in the De Falco et al. (De Falco et al. 2018c) works.

#### Effect of conditioner, spin speed, and washing duration

**Fig. 5:**
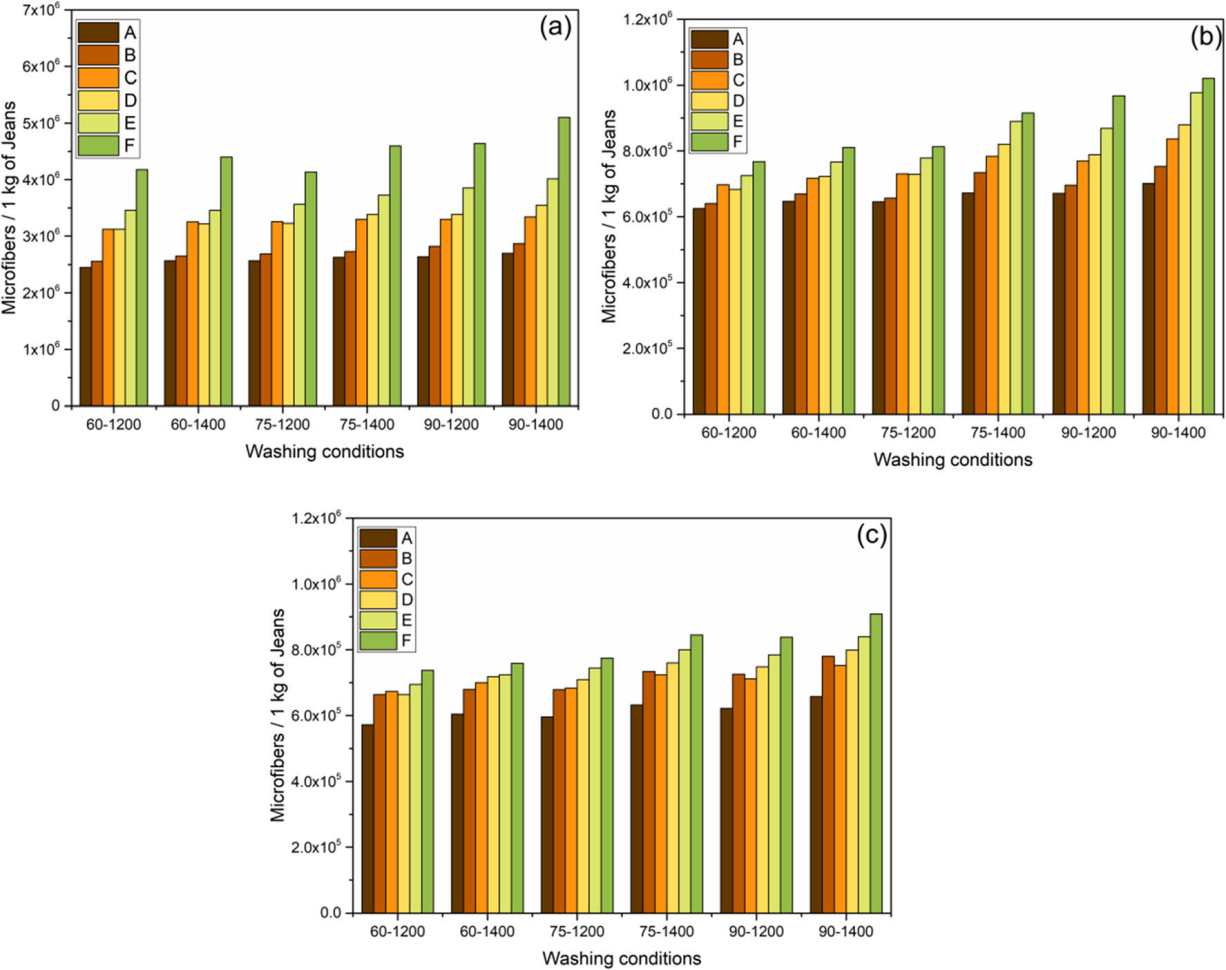
Influence of spin speed and washing duration on microfiber emission, jeans-P (**a**); jeans-PA (**b**); and jeans-PB (**c**) (i.e., washing temperature 45°C)

There were additionally some noteworthy impacts of conditioner use, where all jeans reliably shed more microfibers when conditioner was used as compared to the detergent. In the washing treatment ‘A’ (i.e. spin-speed- 1200rpm, 30°C, 60min) releases 2305395 ± 115269 microfibers per kg of jeans-P (Figure 2-a), (whereas jeans-PA- 619581 ± 30979 and jeans-PB 561683 ± 28084), whereas with the addition of conditioner 2469868 ± 123493, (for jeans-PA- 624039 ± 31201 (Figure 3-a) and jeans-PB 561683 ± 28084 (Figures 4-a and S3)). The presence of detergent and the conditioner play a vital role; it increases the microfiber shedding for jeans-P from 2877278 to 2982681 (i.e., washing treatment C to D) for liquid detergent and 3275154 to 3743691 (i.e., washing treatment E to F) for powder detergent ((Fig. 2a). This trend is almost the same for jeans-PA (Fig. 3a) and jeans-PB (Fig. 4a). Generally, the microfiber releases are the same with washing treatment “C” which linearly increases when increasing the washing durations (Figure. S4). For three jeans, the influence of spin speed with different washing was plotted in Fig. 5. Likewise, the washing duration and the spin speed are directly proportional to the microfiber shedding. This is due to the higher beating and rubbing (friction) action during the washing process. The observed trend is similar for jeans-P (Fig. 5a), jeans-PA (Fig. 5b), and jeans-PB (Fig. 5c). Other than that, it is important to note that the conditioner does not have enough softener content (i.e., hand feel on the jeans after washing); usually, the higher softener content reduces the friction between the machine action and garments during the washing process. In our case, the conditioner manufacturer did not provide the ratio of the chemical composition of the conditioner due to the copyrights and patent. Henceforth, further discussion is not feasible. In jeans-PB, there is 27.5% of microfiber shedding when the temperature increased from 30 to 60°C (Fig. 4c). Additionally, the study nourishes the fact that microfiber emission is influenced by the spin speed. When the spin speed of 1400 rpm with an increase of duration to 90 min shows the rigorous level of microfiber releases, it can be seen in Fig. 2d and e, respectively, for jeans-P and Fig. 4d and e for jeans-PB.

#### Yarn and Fabric characteristics

**Fig. 6:**
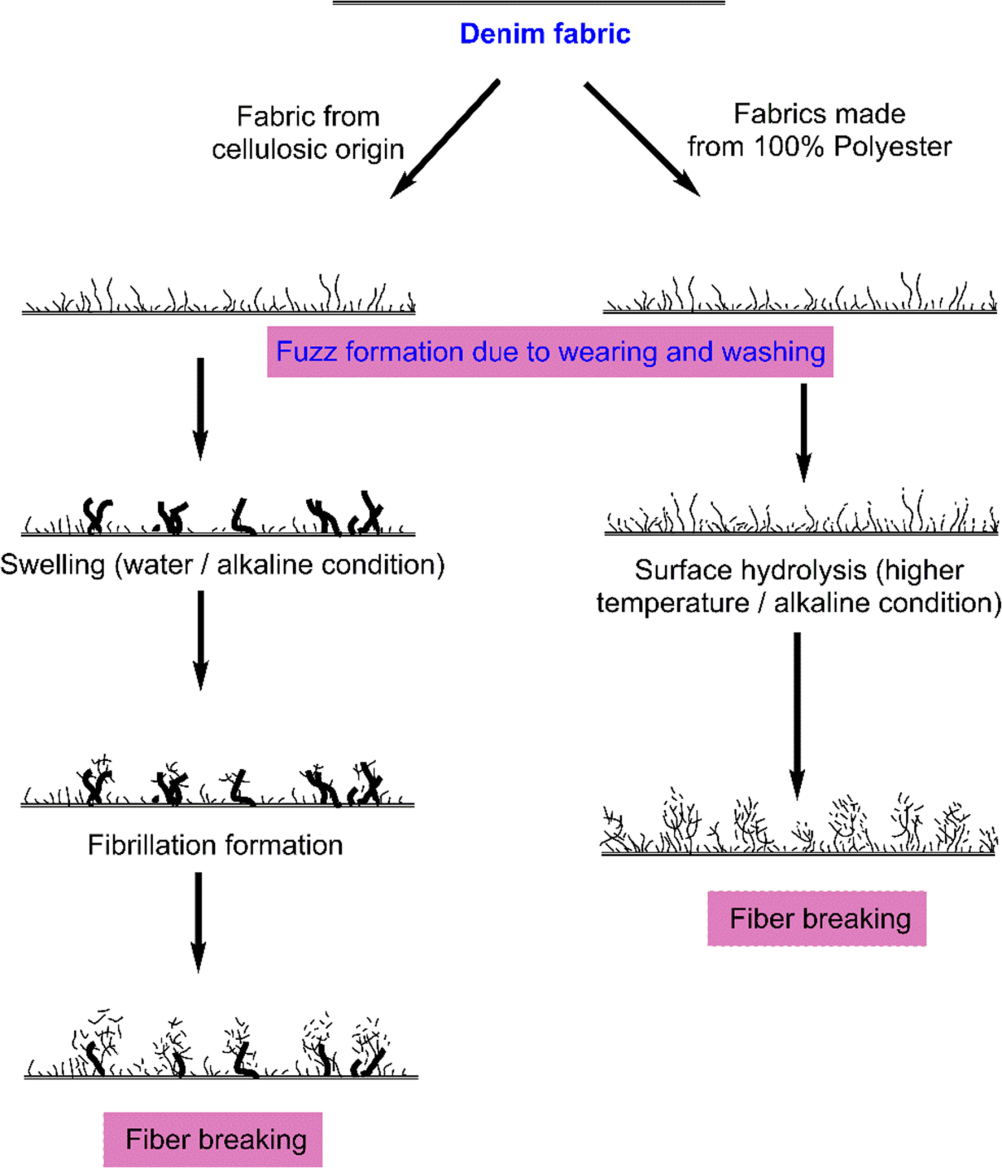
A proposed mechanism of microfiber generation on jeans made from PET and cellulosic fibers (Adapted and modified from (Periyasamy 2020))

Generally, the fabric types (i.e., weave structure and designs) and yarn types (i.e., filament or staple yarns) have a significant influence on the releasing of microfibers during the washing process. Nevertheless, fabrics made from 100% synthetic or synthetic blends release higher microfibers as compared to the natural fibers (Pirc et al. 2016; Napper and Thompson 2016; Sillanpää and Sainio 2017) since our results also confirm it. Moreover, the release of microfibers mostly depends on their blend proportion (Napper and Thompson 2016; Zambrano et al. 2019). The results from the previous works (Napper and Thompson 2016) are more generalized with the microfiber generations and their blend proportion ratio. However, it cannot generalize the releases of microfibers from the fabric made from different blend proportions or 100% synthetic fabric. Yet the following factors such as filament or short-staple fiber, staple length, yarns twist per inch, type of spinning (i.e., open-end, ring spinning), type of yarn (i.e., combed yarn, carded yarn), weave structure, fabric porosity, fabric density (i.e., both warp and weft density), singeing process, number of wet processes, weaving speed, etc. play a vital role in the generation of microfibers. The release of microfiber is higher when the protruding fibers (i.e., due to yarn hairiness) are present in the fabric surface, which is further possible to form the fuzz (Ratnam 2010). The tendency of pilling is initiated from the fuzz formation (Periyasamy 2020) which detaches during the washing process in the machine due to their mechanical action. As per the reference (Chiweshe and Crews 2000), the fabric conditioners increase the possibilities of pilling generations on synthetic fabrics. The pill formation delivers a similar mechanism for both synthetic fibers like polyester and cellulosic fibers like cotton/lyocell; the predicted mechanism for the microfiber generations is shown in Fig. 6. The formation of microfiber and the release is mainly due to the surface rupture of staple fibers under mechanical actions.

In this study, both the warp (doubled) and weft (doubled) for jeans-PA and jeans-PB have the same hairiness index (i.e., Uster hairiness index 3.8, for jeans-P is 4.3) as provided by the producer. The jeans-P has a higher hairiness index than the jeans-PA/jeans-PB (i.e., according to SITRA norms (Ratnam 2010)), which is another reason for the shedding of higher microfibers during the washing. Since hairiness plays a vigorous role (Ratnam 2010); however, it cannot be generalized. In general, hairiness is mostly observed in the yarn made from staple fibers, since the higher cut length may have possibilities of lower hairiness and vice versa.

Frequently, the lengths of staple fiber are usually cut higher than 32 mm either for synthetic or regenerated fibers (Mcloughlin and Hayes 2013). Fiber length is one of the important parameters on the microfiber generations; the longer yarns have the least possibilities of slip from the yarn structure. Microfiber release also depends on other factors such as fabric weight. In general, heavier fabric releases more count of microfibers than lighter fabrics. When the yarn is twisted higher the slipping of fibers from the yarn structure is less which reduces the microfiber releases following Almroth et al. (Carney Almroth et al. 2018). Particularly, garments made out of highly twisted continuous filaments with low hairiness tend to release fewer microfibers during the washing. Generally, the fabric with higher work of rupture (i.e., resistance towards the abrasion), tensile strength, and low hairiness value shows to be the lowest to possibly form fuzz/fibrillation which further breaks and emits from the fabric that is called microfibers.

The 5-way ANOVA was conducted to know the significant complex interaction between five independent variables (jeans types, washing treatment levels (A–F), washing temperature, spin speed, and washing duration); the results are given in Table 4. The possible interaction between the variables is taken into account. The main effects of each variable were consistently significant indicating the variance in microfiber emissions. The interaction effect between the variables (Washing duration * spin speed * washing treatments * washing temperature * jeans types) was significant implying that there is an effect of variables influencing the shedding of microfibers. There is no significant interaction between washing duration * spin speed * washing treatments * washing temperature and spin speed * washing treatments * washing temperature * jeans types. Hence, the highest microfibers were released under washing treatment “F,” 1400 rpm, 90 min, and 60 °C. A list of previous works was summarized in Table S3. For better comparison, our results have been converted according to the previous works. Table S3 shows the perfect comparison of the mass of jeans (garments) rather than the volume of garments. Overall, the comparison states that the number of microfibers released on our results is higher than the previous results.

**Table 4:** Analysis of variance (ANOVA) for factors influencing the shedding of microfibers (Duration, washing duration; speed, spin speed; Temp, washing temperature; and fab, jeans types)

Source	Type III sum of squares	df	Mean square	F	Sig.
Corrected model	525407692641103.2^a^	275	1910573427785	531.987	.000
Intercept	832817750114045	1	832817750114045	231893.7	.000
Duration (60, 75, and 90 min)	780033260034.01	2	390016630017	108.5	.000
Speed (1200 and 1400 rpm)	310658235934.9	1	310658235934.9	86.5	.000
Washing treatments (A-F)	18238594586469.9	5	3647718917293.9	1015.6	.000
Temp (30 °C, 45 °C, and 60°C)	1460583690768.2	2	730291845384.1	203.3	.000
Fab (jeans-P, jeans-PA, and jeans-PB)	482541916568759	2	241270958284379	67180.6	.000
Washing treatments * fab	19153221115388.4	10	1915322111538.8	533.311	.000
Speed * washing treatments	49110020083.1	5	9822004016.6	2.735	.030
Washing treatments * temp	239735000390.4	10	23973500039	6.675	.000
Duration * washing treatments	95802979982	10	9580297998.2	2.668	.011
Speed * washing treatments * fab	169956238685.1	12	14163019890.4	3.944	.000
Washing treatments * temp * fab	1318040351635.4	12	54918347984.8	15.2	.000
Duration * washing treatments * fab	228483146982.7	24	9520131124.2	2.6	.002
Speed * washing treatments * temp	51635799971.8	12	4302983330.9	1.19	.312
Duration * speed * washing treatments	148150835645.3	12	12345902970.4	3.43	.001
Duration * washing treatments * temp	87219439823.5	12	3634143325.9	1.01	.471
Speed * washing treatments * temp * fab	74705070288.9	24	3112711262	.867	.640
Duration * speed * washing treatments * fab	176525950365.8	24	7355247931.9	2.048	.017
Duration * washing treatments * temp * fab	218677099466.7	48	4555772905.5	1.269	.206
Duration * speed * washing treatments * temp	64643250429.6	24	2693468767.9	.750	.775
Duration * speed * washing treatments * temp * fab	1823859458646	48	390016634455	.685	.412
Error	172386105304.8	24	3591377193.8		
Total	1358397828860285	324			
Corrected Total	525580078746408	323			

a. R Squared = 1.000 (Adjusted R Squared = .998)

## Conclusion

The current study aimed to quantify the microfiber emission during domestic washing of jeans with varied washing conditions. From this work, it is concluded that the jeans types, washing temperature, washing duration, spin speed, detergent types, and addition of conditioner strongly influence the microfiber generations. When washing undergoes domestic treatments, the results show that jeans-P releases the highest number of microfibers concerning jeans-PA and jeans-PB. Further trials confirmed that the powder detergent sources are higher than the generation of microfibers as compared to liquid detergent. The approximate number of microfibers released from a typical 1 kg wash load of jeans-P was calculated in the range of 2305395–4874323.3 (for jeans-PA 619581.3–1085431.3 and 561683.2–865182.1 for jeans-PB) as it is influenced by the washing treatments. There was a consistent trend between the jeans types. A 5-way analysis of variance (ANAVO) reveals significant interactions between 5 variables. To prevent microfiber pollution, it is essential to consider the factors in the calculation of risk reduction interventions. Henceforth, it is mandatory to extend this work to have detailed data regarding the types of washing machines, age of jeans, and fabric weave structure; however, be beneficial. Microfiber generations are also influenced by the physical and chemical properties of fibers, yarn, and fabric apart from the washing treatments. Hence, this investigation refurbishes the jeans industry to contribute less microfiber emission.

## Supplementary Information

ESM 1 (DOCX 12.4 mb)
