# Supplementary material for: Evaluation of microfiber release from jeans: the impact of different washing conditions
Source: Environ Sci Pollut Res Int. 2021 Jun 11;28(41):58570–82. doi: 10.1007/s11356-021-14761-1 (PMC8536618; doi:10.1007/s11356-021-14761-1)
Supplement: Supplementary file 1 — (DOCX 12.4 mb) [file 11356_2021_14761_MOESM1_ESM.docx]

**Supporting information**

**Evaluation of microfiber release from jeans: the impact of different washing conditions**

**Aravin Prince Periyasamy**

Textile Chemistry, Department of Bioproducts and Biosystems, School of Chemical Engineering, Aalto University, Espoo, 02150, Finland.

E-mail: [aravinprince.periyasamy@aalto.fi](mailto:aravinprince.periyasamy@aalto.fi)

In this work, the inclusion and exclusion criteria are given in the Table S1. In fact, the exclusion criteria may help to work further.

**Table S1.** Inclusion and exclusion criteria for the plan of work

| Inclusion criteria | Exclusion criteria |
| --- | --- |
| Washing duration, washing temperature, washing spin-speed, detergent (mild and heavy), conditioner and blends of synthetic fibers. | Age of garment, washing machine type (front / top load), fabric weave structure. |

***Cleaning of washing machine***

Prior to jeans washing, the machines were cleaned two times using rigorous settings (120 min, 60 °C, 1400 rpm). The addition of citric acid (200 grams) was added during the first cycle of cleaning and monitor the machine filter to ensure there is no microfibers and other contaminations in the filters. This makes clear that the machine is clean. The machine was cleaned thoroughly after each utilization of jeans sample washing as explained above.

***Filtration***

The stainless-steel filter (diameter 33.4 mm; pore size-200 µm × 200 µm) was purchased from Bolin metal wire mesh co., ltd, P.R. China, for PTFE filter (diameter 33.4 mm; pore size-5 µm × 5 µm) was purchased from Cole-Parmer India.

***Hairiness Measurement***

The yarn was raveled from the jeans and tested in Uster hairiness tester (Uster Standard Test Method) under ASTM standard conditions. SITRA (Ratnam 2010) norms was used for the data comparison.

**
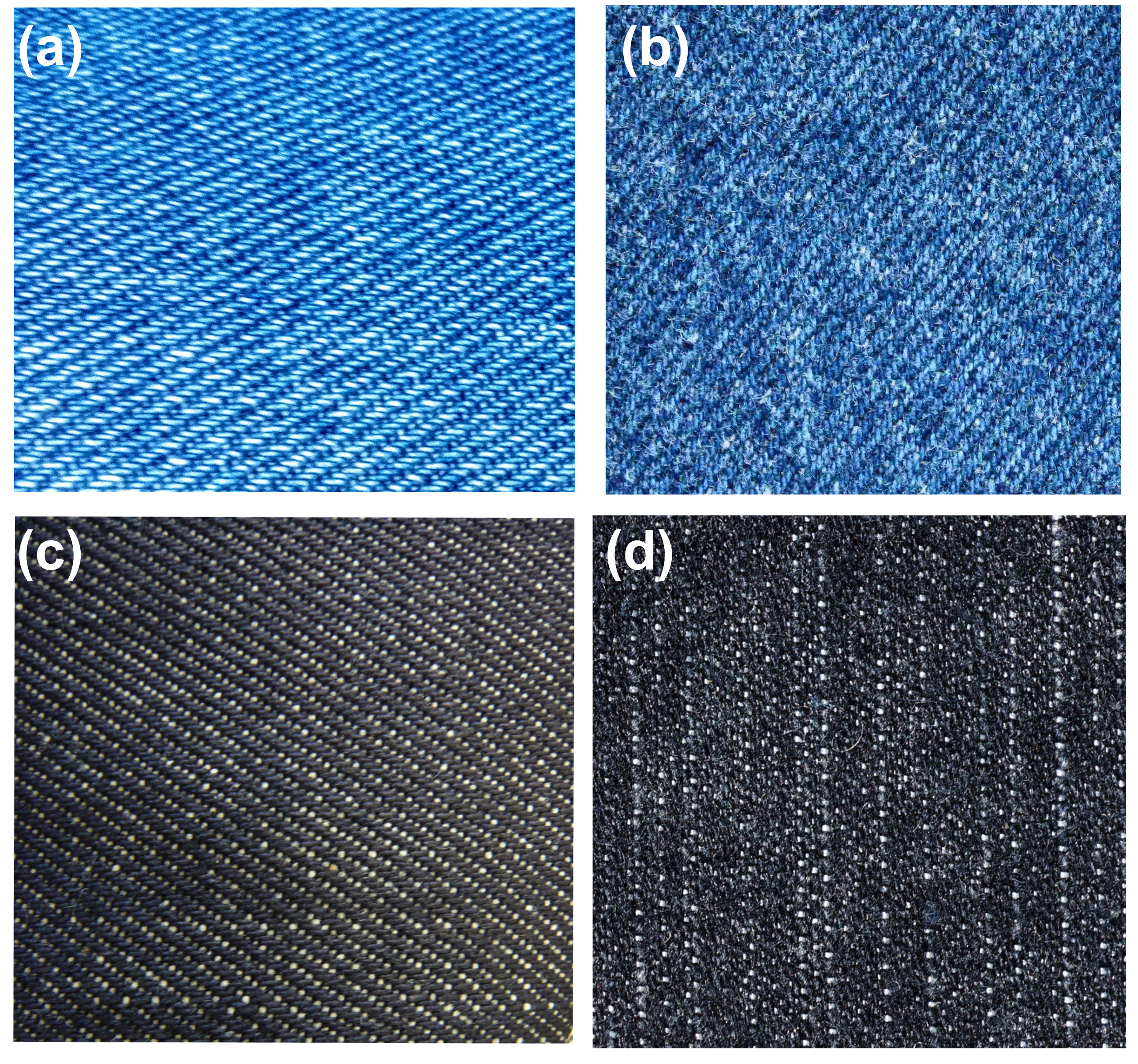
**

**Figure S1.** Typical jeans used for this study unwashed jeans-P (a); jeans-P washed with powder detergent/conditioner (b); unwashed jeans-PB (c); jeans-PB washed with powder detergent/conditioner (d).

**Table S2.** The composition of different detergent used for this study.

| ***Liquid detergent*** | Wt. % | ***Powder detergent*** | Wt. % |
| --- | --- | --- | --- |
| Water | 65 | Sodium sulfate | 25.35 |
| Alkyl(C12-14) diglykoletherfulfate, Na-salt | 6 | Sodium hydroxide | 0.40 |
| n-Alkyl(C10-13) benzolsufonic acid | 5 | Sodium carbonate | 20-25 |
| 1,2-Propyleneglycol | 4-11 | Sodium-alluminim silicate (Zeolite A) | 10-15 |
| Alkyl(C12-18) carbonic acid | 4-5 | Sodium silicate | 10 |
| Oxoalcohol(C_13_-_15_) polyetheylenglycolether | 3-8 | Tetraacethylethylenediamine | 7-8 |
| Sodium hydroxide | 1-1.5 | Sodium methyl 2-  sulphooctadecanoate and soldium  1-methoxy-1-oxohexadecane-2-  sulphonate | 8 |
| Trisodiumsulfate dihydrate | 2 |  |  |
| Scents | 0.50 | Scent | 0.5 |
|  |  | Titanium dioxide | 0.5 |
|  |  | Copolymer with 1-Vinylmidazol  and 1-Vinyl-2-pyrrolidine | 0.2 |
| Brand | Ariel | Ariel | |
| pH | 9.1 | 10.6 | |
| Presence of inorganic compounds | -no data- | Yes, Titanium pigments | |
| Conditioner (Lenor) | Conditioner with cationic surfactant, scents and silicones. | | |

***FTIR characterization***

Considering the jeans-PA, the sharp peaks at 3323 confirms the NH stretching, 1734 confirms the C=O of lycra portions (Figure S2-a/b) the similar peaks can be observed in the jeans-P (Figure S2-e/f). Additionally, the jeans-PA and jeans-PB has similar spectra except lycra segments. For jeans-PB, 3280 cm^-1^ (O-H stretching band), 2920 cm^-1^ (CH_2_ asymmetrical stretching’s), 2850 cm^-1^ (CH_2_ symmetrical stretching), 1713 cm^-1^ (C=O stretching, i.e. resembling polyester (PET) portion), 1640 cm^-1^ (O-H bending), 1335 cm^-1^ (C-H bending), 1314 cm^-1^ (CH_2_ rocking), 1245 cm^-1^ (ester C-O stretching) 1110 cm^-1^ (C-O-C stretching), 1090 cm^-1^, 1056 cm^-1^, 1033 cm^-1^, 1000 cm^-1^ and 985 cm^-1^ (C-O stretch) and 720 cm^-1^ (C-H aromatic ring) (Ueland et al. 2017) confirming the observed peaks as polyester cotton blends (Figure S2- c/d). In the spectra 2969 cm^-1^, 2907 cm^-1^ (C-H stretching), 1711 cm^-1^ (C=O vibrations), 1504 cm^-1^, 1409 cm^-1^ (aromatic ring C=C stretching), 1339 cm^-1^, 1241 cm^-1^ (carboxylic ester), 1093 and 1014 (O-C-O stretching), 971 cm^-1^ (C=C), 871 cm^-1^ (aromatic ring C-H bending), 847 cm^-1^ (two neighbouring H in benzene), and 724 cm^-1^ (C-H aromatic ring) confirms the jeans-P (Figure S2- e/f).





**Figure S2.** ATR-FT-IR spectrum of samples, (a) jeans-PA before washing; (b) microfibers from jeans-PA after washing; (c) jeans-PB before washing; (d) microfibers from jeans-PB after washing; (e) jeans-P before washing; (f) microfibers from jeans-P after washing.

***Effect of conditioner on microfiber releases***


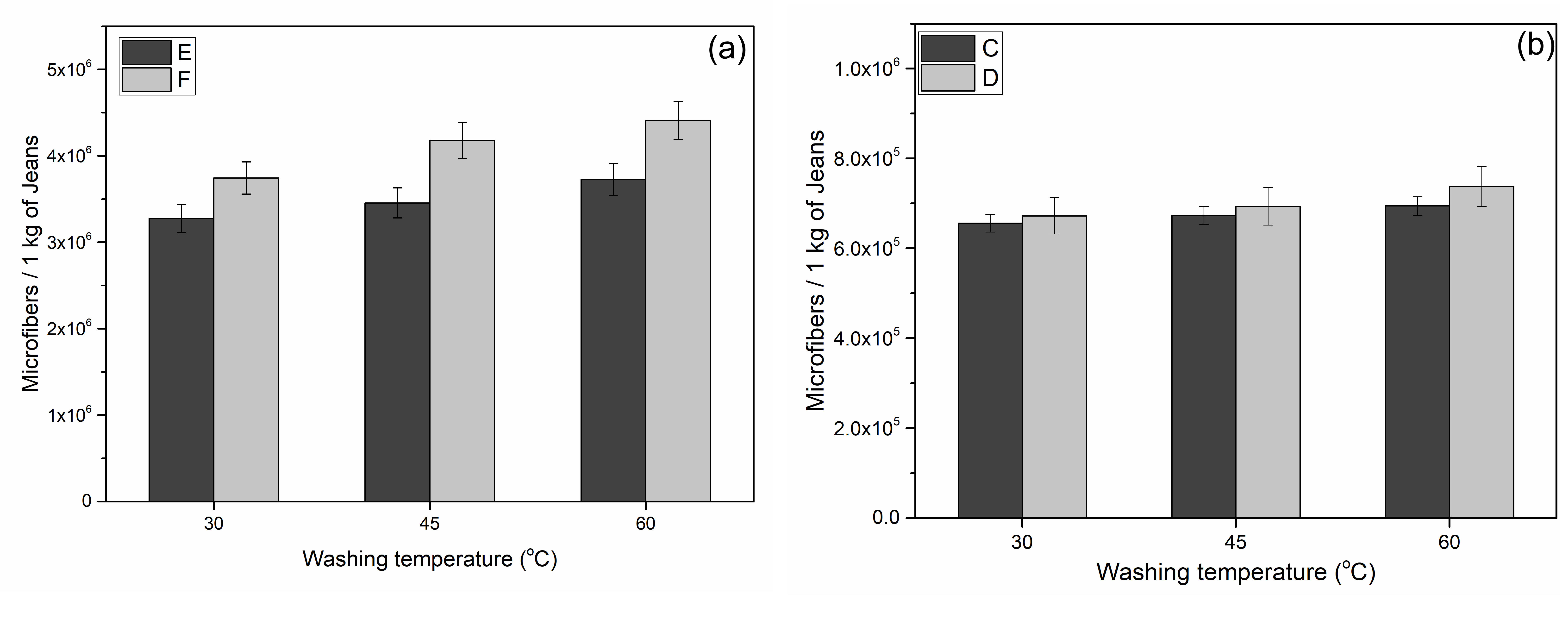


**Figure S3.** Effect of conditioner on microfiber emission for jeans-P (a) and jeans-PB (b) (i.e. washing duration 60 mins / spin-speed 1200 rpm).

***Effect of washing duration on microfiber releases***





**Figure S4.** Effect of washing duration on microfiber emission (washing temperature -45 °C spin-speed 1200 rpm).

**Table S3.** Previous published works information and comparisons with the present work.

| Authors | Research Work & Materials | Results and outcome | Ref | Our results |
| --- | --- | --- | --- | --- |
| Harline et al. | 100 % PET | 449 ± 100, 122 ± 100 mg of microfibers per garments in top and front-loading washing machines respectively | (Hartline et al. 2016) | 1.923 - 3.485 mg of microfibers per jeans-P garment;  0.335 – 0.488 mg of microfibers per P/C jeans garment |
|  | Recycled PET/ virgin PET (85%:15%) | 526 ± 129, 25 ± 22 mg of microfibers per garments in top and front-loading washing machines respectively |  |  |
|  | 100 % nylon | 333 ± 100, 69 ± 100 mg of microfibers per garments in top and front-loading washing machines respectively |  |  |
|  | Recycled PET/virgin PET/spandex (63%:33%:3%) | 538 ± 419, 35 ± 29 mg of microfibers per garments in top and front-loading washing machines respectively |  |  |
| Falco et al. | 100 % PET (woven and knitted), PET/cotton (50%:50%) | Woven 100 % PET releases 128 ± 62 mg of microfibers per kg fabric.  Knitted 100 % PET releases 296 ± 36 mg of microfibers per kg fabric | (De Falco et al. 2020) |  |
| Napper and Thompson | PET/cotton (65%:35%), 100 % PET and Acrylic garments used. | 140000-730000 microplastics/ 6kg of washed garments | (Napper and Thompson 2016) | 13832371 – 29245940 microfibers per 6 kg of 100 % polyester garment;  3370098 – 5191093 microfibers per 6 kg of polyester/cotton garment |
| Falco et al.* | 100 % PET (woven and knitted) and polypropylene | 972000-21228000 microfibers /6 kg of washed garments | (De Falco et al. 2018) |  |
| Pirc et al. | 100 % PET fleece blankets | 135000 microfibers/6 kg of washed garments | (Pirc et al. 2016) |  |
| Belzagui et al. | 100 % PET, PET/elastane (80%:20%) and acrylic/polyamide (70%:30%) |  | (Belzagui et al. 2019) |  |
| Falco et al. | 100 % PET, recycled PET | 640000-1500000 microfibers/ washed one single garment | (De Falco et al. 2019) | 11037427 - 2193445 of microfibers per single 100 % PET jeans garment;  252757 - 389331 of microfibers per single P/C jeans garment |

* for better comparisons, our results are converted into 6kg of washed garments.

From the Table S3, if relating to the number of microfiber emission, our findings are often higher than prior publications (Ref. (Pirc et al. 2016); (De Falco et al. 2018); (De Falco et al. 2018) and (Belzagui et al. 2019)), while in some situations they are identical (Ref. (De Falco et al. 2019)). For certain instances, our findings are lower (Hartline et al. 2016) attributable to certain other factors, in particular the type of fabric and their washing treatments.

***Microfibers from domestic washing versus nations***

Additionally, the scope of this investigation can be further expanded to examine the emission of microfibers in other countries together considering the washing cycles according to the wearing cycles. It is to be noted that the USA consumers jeans per capita is eight pairs whereas for France/UK, China and India is 3, 1 and 0.3 pair respectively per year according to CIA factbook (2019). The mean washing frequency of various nations is been studied by the Levi’s for their Life cycle assessment of jeans (Levi Strauss & Co. 2015). In the various countries, Chinese consumers wearing jeans 3.9 times for one washing cycle, 2.3 and 2.5 for consumers from USA & UK/France respectively. Roughly, the French consumers can wash their jeans between 20 to 50 times per year (Levi Strauss & Co. 2015).

It is surprise to check their washing frequency versus the microfiber generations, results the mass of released fibers tends 1.92 - 3.48 mg for single jeans-P and 0.33 - 0.478 mg for the single jeans-PB. The microfibers exit annually per Jeans is based on the obtained results were 96.1 mg for jeans-P and 16.5 mg for jeans-PB under room temperature washing and without any detergent/conditioners. Similarly, in the powder detergent with conditioner environment, the jeans-P 60 min emits 157.8 mg and 90 min emits 168.4 mg of microfibers. This result is applicable for single garment, it is very difficult to predict the actual numbers since the average French customers purchase 3 jeans per year. However, it is just a hypothetical calculation and the main motto to create awareness to the jeans wearers. In order to evaluate scientifically the overall emissions of microfibers from the separate nations, it is essential to require the following data;

- There is no deep data for purchasing of polyester jeans.
- No proper data for washing or washing / drying (tumble drying)
- If they purchase all denims/jeans made from 100 % polyester or polyester:cotton (50:50), then the above calculation is feasible.

The proportion reaches to metric tons approximately in a nation according to the conservative estimate since an average person may also own other synthetic textiles and other kinds of stuff like blankets, carpets, sportswear, gloves, caps, shopping bags, PET bottles, etc made of synthetic fibers.

**Reference**

Belzagui F, Crespi M, Álvarez A, et al (2019) Microplastics’ emissions: Microfibers’ detachment from textile garments. Environ Pollut 248:1028–1035. https://doi.org/10.1016/j.envpol.2019.02.059

De Falco F, Cocca M, Avella M, Thompson RC (2020) Microfiber Release to Water, Via Laundering, and to Air, via Everyday Use: A Comparison between Polyester Clothing with Differing Textile Parameters. ACS Appl Mater Interfaces 54:3288–3296. https://doi.org/10.1021/acs.est.9b06892

De Falco F, Di Pace E, Cocca M, Avella M (2019) The contribution of washing processes of synthetic clothes to microplastic pollution. Sci Rep 9:6633. https://doi.org/10.1038/s41598-019-43023-x

De Falco F, Gullo MP, Gentile G, et al (2018) Evaluation of microplastic release caused by textile washing processes of synthetic fabrics. Environ Pollut 236:916–925. https://doi.org/10.1016/j.envpol.2017.10.057

Hartline NL, Bruce NJ, Karba SN, et al (2016) Microfiber Masses Recovered from Conventional Machine Washing of New or Aged Garments. Environ Sci Technol 50:11532–11538. https://doi.org/10.1021/acs.est.6b03045

Levi Strauss & Co. (2015) Understanding the environmental impact of a pair of Levi’s ®501 ®

Napper IE, Thompson RC (2016) Release of synthetic microplastic plastic fibres from domestic washing machines: Effects of fabric type and washing conditions. Mar Pollut Bull 112:39–45. https://doi.org/10.1016/j.marpolbul.2016.09.025

Pirc U, Vidmar M, Mozer A, Kržan A (2016) Emissions of microplastic fibers from microfiber fleece during domestic washing. Environ Sci Pollut Res 23:22206–22211. https://doi.org/10.1007/s11356-016-7703-0

Ratnam TV (2010) SITRA Norms for Spinning Mills. South India Textile Research Association, Coimbatore, India

Ueland M, Howes JM, Forbes SL, Stuart BH (2017) Degradation patterns of natural and synthetic textiles on a soil surface during summer and winter seasons studied using ATR-FTIR spectroscopy. Spectrochim Acta Part A Mol Biomol Spectrosc 185:69–76. https://doi.org/10.1016/j.saa.2017.05.044

(2019) CIA Factbook. In: CIA Factb. https://www.cia.gov/library/publications/resources/the-world-factbook/. Accessed 28 Mar 2020
